# Psychotropic medication use among nursing home residents in Austria: a cross-sectional study

**DOI:** 10.1186/1471-2318-9-18

**Published:** 2009-05-21

**Authors:** Eva Mann, Sascha Köpke, Burkhard Haastert, Kaisu Pitkälä, Gabriele Meyer

**Affiliations:** 1 General practice and Institute for Health Services Research, Rankweil, Austria; 2 Unit of Health Sciences and Education, University of Hamburg, Hamburg, Germany; 3 mediStatistica, Neuenrade, Germany; 4 Department of General Practice and Primary Health Care, Unit of General Practice, Helsinki University Hospital and Faculty of Medicine, University of Helsinki, Helsinki, Finland; 5 Institute of Nursing Science, Faculty of Medicine, University Witten/Herdecke, Witten, Germany

## Abstract

**Background:**

The use of psychotropic medications and their adverse effects in frail elderly has been debated extensively. However, recent data from European studies show that these drugs are still frequently prescribed in nursing home residents. In Austria, prevalence data are lacking. We aimed to determine the prevalence of psychotropic medication prescription in Austrian nursing homes and to explore characteristics associated with their prescription.

**Methods:**

Cross-sectional study and association analysis in forty-eight out of 50 nursing homes with 1844 out of a total of 2005 residents in a defined urban-rural region in Austria. Prescribed medication was retrieved from residents' charts. Psychotropic medications were coded according to the Anatomical Therapeutic Chemical Classification 2005. Cluster-adjusted multiple logistic regression analysis was performed to investigate institutional and residents' characteristics associated with prescription.

**Results:**

Residents' mean age was 81; 73% of residents were female. Mean cluster-adjusted prevalence of residents with at least one psychotropic medication was 74.6% (95% confidence interval, CI, 72.0–77.2). A total of 45.9% (95% CI 42.7–49.1) had at least one prescription of an antipsychotic medication. Two third of all antipsychotic medications were prescribed for bedtime use only. Anxiolytics were prescribed in 22.2% (95% CI 20.0–24.5), hypnotics in 13.3% (95% CI 11.3–15.4), and antidepressants in 36.8% (95% CI 34.1–39.6) of residents. None of the institutional characteristics and only few residents' characteristics were significantly associated with psychotropic medication prescription. Permanent restlessness was positively associated with psychotropic medication prescription (AOR 1.54, 95% CI 1.32–1.79) whereas cognitive impairment was inversely associated (AOR 0.70, 95% CI 0.56–0.88).

**Conclusion:**

Frequency of psychotropic medication prescription is high in Austrian nursing homes compared to recent published data from other countries. Interventions should aim at reduction and optimisation of prescriptions.

## Background

Several studies have shown high prevalences of psychotropic medications in frail elderly people [1-4]. Nursing home residents represent a frail population, requiring special attention on adverse drug reactions due to multiple drug use, combined with age-related pharmacokinetic and pharmacodynamic changes [5].

Reported prevalence of psychotropic medications in nursing homes varies substantially between studies with a range of 50% to 80% of residents with at least one psychotropic medication prescription, depending on the setting, country, and cultural background [2].

In Europe, psychotropic medications are often used to control behavioural and psychological symptoms of dementia (BPSD). A meta-analysis found that atypical antipsychotics were the only psychotropic medications that were effective in the treatment of BPSD [6]. However, their effectiveness is restricted to aggression, agitation and psychotic symptoms. The effects are moderate and may be offset by severe adverse events [7,8].

Sedation, falls, extrapyramidal and anticholinergic symptoms are well-described adverse effects of psychotropic medication [9,10]. Atypical and typical antipsychotics may increase the risk of stroke and death [11-13], although results are contradictory [12,14,15]. Benzodiazepines and antidepressants increase the risk of falls and fractures [9,16,17].

Study results on determinants of psychotropic medication prescription in nursing home residents are conflicting. Associations between psychotropic medication prescribing and individual residents' characteristics as age, gender, and medical conditions as well as institutional characteristics show substantial variations throughout countries and within the same country [2,18-21].

So far, prevalence data on psychotropic medication prescription in Austrian nursing homes are lacking. According to a recent legal act in Austria, the so-called "Heimaufenthaltsgesetz" [22], nursing homes are obliged to report not only physical restraints but also chemical restraints. Unfortunately, no national guideline defines inappropriate prescriptions, which could act as chemical restraint [23].

Descriptive data on routine care are a prerequisite to optimise reporting of inappropriate prescription and to shape future interventions to reduce psychotropic medication in nursing home residents. Since many reports on adverse effects of psychotropic medications have recently been published, we assumed that prescription frequency would be lower in Austria than reported in earlier prevalence studies from other countries [2]. We aimed to determine the prevalence of prescribed psychotropic medication and to explore associations between psychotropic medication prescription and institutional and residents' characteristics.

We hypothesised psychotropic medication prescription to be higher in cognitively impaired and more care-dependent residents and lower in residents with dementia but without concomitant behavioural disturbances. We also assumed higher psychotropic prescription rates in nursing homes with a lower resident/staff ratio.

## Methods

### Nursing homes and residents

We invited all 50 nursing homes providing 2005 nursing home beds in the federal state of Vorarlberg, Austria, to participate. Vorarlberg is an urban-rural region in the most Western part of Austria. A total of 48 out of 50 nursing homes agreed to participate. Data collection took place from March 2007 to September 2007. Three trained medical doctors and one trained advanced medical student performed data collection. The training was provided by the principal investigator (EM). It covered a three-hour personal instruction about psychotropic medication use in the elderly and provision of a written instruction manual on the application of the study's data collection sheets. The study population consisted of all residents who were present in the nursing home at the day of data collection. All medications, which were prescribed and administered at least once per day were retrieved from the residents' charts by nursing staff and documented by the trained assessors. Data on medication prescribed "as required" were not included. Two research assistants at the University of Hamburg coded all medications prescribed according to the WHO Anatomical Therapeutic Chemical Classification (ATC 2005) [24]. Psychotropic medications were categorised as: antipsychotics (ATC-Category N05A), anxiolytics (N05B), hypnotics (N05C) and antidepressants (N06A).

For data protection reasons, we were unable to assess residents' functional status. Therefore, we chose using the levels of long-term care need as assessed by trained physicians on behalf of the Austrian Federal Act on Nursing Care (Bundespflegegesetz, 1993) [25]. Since 2003, this assessment has been administered to each Austrian citizen requiring statutory offered nursing care. Level 1 is related to a monthly amount of nursing care time of ≥ 50 to 75 hours, level 2 to ≥ 75 to 120 hours, level 3 to ≥ 120 to 160 hours, and level 4 to ≥160 to 180 hours. For and beyond level 5 additional care is needed due to blindness or deafness, permanently required surveillance, or complete immobility.

Information on history of falls and fractures was retrieved from residents' charts.

Cognitive status was determined using the Dementia Screening Scale (DSS) [26], a validated eight-question proxy-rating screening tool for use by nursing staff. The items address personal, temporal, and local orientation during the last four weeks. The optimal cut-off level indicating relevant cognitive impairment has been defined as ≥ 4 points revealing a sensitivity of 89% and a specificity of 87% for moderate to severe dementia. An advantage of the DSS is that the number of missing cases is lower than in the cognitive tests commonly used, e.g. Mini-Mental State Examination or the Dementia Scale of the Brief Assessment Schedule.

Residents' behavioural and psychological symptoms of dementia (BPSD) were determined using an abbreviated Cohen Mansfield Agitation Inventory [27], comprising five items on the frequency of behavioural symptoms during the preceding 4 weeks. Nurses who knew the residents well were asked to rate frequency of general restlessness, verbal agitation, handling things inappropriately, negative attitude, and aggression on a four-point Likert scale (never, once or twice, repeatedly, permanently).

Data collection sheets and procedure have been successfully tested within a recently conducted study in Germany [3]. Data entry and coding were double checked by an independent research assistant. The ethics committee of the federal state Vorarlberg approved the study protocol.

### Statistical methods

Baseline characteristics of nursing homes and residents were described as means ± standard deviations (SD), and numbers and percentages. Cluster-adjustment of these data was avoided in order to describe the raw baseline characteristics of the study population. A cluster was defined as a nursing home. All parameters describing psychotropic medication were considered as outcomes. These outcome variables are correlated within the clusters. Since data were collected across a number of clusters, the fact that each resident of a nursing home has common influences means that each individual's data must be adjusted in order to obtain a reliable estimate of effect size and precision. Methods for cluster-adjusted estimation of prevalence, means and their variances are well known from cluster-randomised trials [28,29] and are also recommended for non-randomised trials [28]. Estimators describing outcomes (e.g. prescription prevalence) were calculated as weighted means over all clusters. Minimum variance weights were used instead of the frequently used cluster size weights because of their advantages in case of unequal cluster sizes [29]. For each outcome variable the cluster correlation was estimated by the corresponding intracluster correlation coefficient (ICCC).

From the cluster-adjusted estimators cluster-adjusted approximate two-sided 95% confidence intervals (CIs) and cluster-adjusted standard deviations (SDs) were calculated.

Associations of characteristics of institutions or residents with prescription of psychotropic medication were investigated by multiple logistic regression analysis. Correlation within the clusters was considered by robust variance estimation [30,31]. Cluster-adjusted odds ratios (AORs) were estimated in these models.

Logistic regression models were fitted separately with regard to five different dependent variables: prescription of any psychotropic medication and prescription of antipsychotic, anxiolytic, hypnotic or antidepressive medication.

The following characteristics of institutions and residents were considered as independent variables: Age, length of stay in the nursing home, short time nursing care, level of long-term care need, legal guardian designated, fall during preceding four weeks, fall during preceding 12 months, fracture during preceding 12 months, permanent restlessness, permanent verbal agitation, permanently handling things inappropriately, permanent negative attitude, permanent aggression, cognitive impairment, ownership of homes, number of residents per cluster (in the study population), number of residents per caregiver, proportion of trained nurses. The logistic model considering each dependent variable was fitted in the following way: At first, each independent variable was evaluated in a univariate model. All co-variables significantly associated with the outcome were included in a multiple model. From this multiple model all non-significant co-variables were deleted. The resulting multiple model was the main model with regard to this dependent variable. Finally, to get unified models for each of the five dependent variables, all independent variables from the five main models were combined in the final logistic regression models, even if the associations were not significant in each model. Results of the final models are presented. Adjustment for multiple testing was not performed, since multiple hypotheses were not tested. Results are interpreted in an explorative manner.

The level of significance was 0.05. Statistical analysis was performed using the statistical software packages SAS 9.2 TS1M0 and STATA 10.0 (robust variance estimation in logistic regression models).

## Results

### Nursing homes and residents

A total of 1844 residents were included. Residents' mean age was 81 years (SD 11.6, range 29–108), 73% were female. Mean length of stay in the nursing home was 52 months (SD 67.2, range 0–571). Residents' characteristics are displayed in Table 1.

**Table 1:** Characteristics of residents.*†

Characteristic	n = 1844
Women	1340 (73)
Mean ± SD (range) age, years	81 ± 12 (29–108)
Legal guardian designated	852 (46)
Level of long-term care need	
0–2	155 (8)
3–5	1031 (56)
6–7	644 (35)
Fall during preceding 12 months	711 (39)
Fracture during preceding 12 months	134 (7)
History of hip fracture	73 (4)
Agitated behaviour^‡^	
Restlessness	
once or twice	233 (13)
repeatedly/permanently	511 (28)
Verbal agitation	
once or twice	171 (9)
repeatedly/permanently	399 (22)
Handling things inappropriately	
once or twice	222 (12)
repeatedly/permanently	363 (20)
Negative attitude	
once or twice	288 (16)
repeatedly/permanently	466 (25)
Aggression	
once or twice	241 (13)
repeatedly/permanently	216 (12)
Cognitive impairment (cut-off ≥ 4)	880 (48)

Values are numbers (percentage) unless stated otherwise.

* Not cluster-adjusted.^† ^In some items figures do not cumulate to the total number of residents investigated due to missing values. ^‡ ^During preceding 4 weeks.

The majority of nursing homes were owned by non-profit organisations (85%), of these 8% were state owned, and 6% affiliated to church. Further 15% were for-profit organisations. A small number of residents lived at a specialised dementia care unit (n = 81, 4.4%) and 53 persons (2.9%) were admitted for intermediate care.

The mean number of residents per home was 38 (SD 21, range 5–113). The mean number of residents per full-time nursing staff was 2.4 (SD 0.82, range 1.53–5.50); 41% (SD 20, range 0–100) of nursing staff were trained nurses.

### Prevalence of psychotropic medication use

Mean cluster-adjusted prevalence of residents with at least one psychotropic medication was 74.6% (95% CI 72.0–77.2). The mean number of psychotropic medications per resident with at least one prescription was 1.88 (95% CI 1.82–1.94).

A total of 45.9% (95% CI 42.7–49.1) had at least one prescription of an antipsychotic medication. At least one typical antipsychotic medication was prescribed in 34.8% (95% CI 31.7–37.9) of residents and an atypical antipsychotic in 19.5% (95% CI 17.2–21.8). The most often prescribed antipsychotic medication was prothipendyl, an azaphenothiazin structurally related to phenothiazines. It was prescribed in 25.9% of residents and in all but one patient for bedtime use. Prothipendyl is an older drug not used in most European countries due to frequent extrapyramidal side effects.

Data on psychotropic medication are displayed in Table 2.

**Table 2:** Prescribed psychotropic medication.

Residents with at least one prescription of ...	n = 1375	ICCC
Any psychotropic medication	74.6 (72.0–77.2)	0.016
Antipsychotic medication	45.9 (42.7–49.1)	0.023
Conventional, low potency	31.5 (28.4–34.5)	0.025
Conventional, middle and high potency	6.4 (5.2–7.7)	0.006
Atypical	19.5 (17.2–21.8)	0.014
Anxiolytic medication	22.2 (20.0–24.5)	0.009
Benzodiazepine	21.6 (19.3–23.9)	0.011
Other	0.9 (0.4–1.3)	0.000
Hypnotic medication	13.3 (11.3–15.4)	0.017
Benzodiazepine	11.4 (9.6–13.3)	0.015
Zolpidem, Zopiclon	2.0 (1.4–2.6)	0.000
Antidepressant medication	36.8 (34.1–39.6)	0.012
Selective serotonin reuptake inhibitor	30.8 (28.2–33.5)	0.013
Tricyclic	8.2 (7.0–9.5)	0.000
Other	2.1 (1.3–2.9)	0.015

Values are cluster-adjusted percentages (95% confidence interval) and intracluster correlation coefficients (ICCC).

### Associations of psychotropic medication with residents' characteristics

We could not find any statistically significant association between psychotropic medication prescription and nursing home characteristics such as number of residents per nursing home (univariately p = 0.406) or the proportion of trained nurses (univariately p = 0.910). The association between the number of residents per caregiver and psychotropic medication use was borderline significant (using three classes: baseline 1.9–2.2 residents per caregiver, <1.9 and >2.2): In the adjusted model including all other covariables of the final models as described in Table 3 and Additional file 1, the AOR of "less than 1.9 residents per caregiver" was 1.52, (95% CI 0.93–2.50, p = 0.097). The AOR of "more than 2.2 residents per caregiver" was 1.43 (95% CI 0.99–2.06, p = 0.057).

**Table 3:** Characteristics associated with psychotropic medication prescription.

Characteristics	AOR (95% CI) n = 1690*, R^2 ^= 0.056	p-value
Age (Years, continuous variable; AOR per 1 year increase)	0.99 (0.98–1.00)	0.025
Male (Reference: female)	0.71 (0.54–0.93)	0.013
Level of long-term care need ≥ 4 (Reference: 0–3)	1.70 (1.28–2.26)	<0.001
Legal guardian designated (Reference: no)	1.10 (0.86–1.41)	0.452
Fall during preceding 12 months (Reference: no)	1.66 (1.26–2.18)	<0.001
Permanent restlessness (ordinal 1–2–3–4, reference: 1 = never; AOR per 1 unit increase)	1.52 (1.30–1.76)	<0.001
Permanently handling things inappropriately (ordinal 1–2–3–4, reference: 1 = never; AOR per 1 unit increase)	0.97 (0.83–1.14)	0.728
Permanent negative attitude (ordinal 1–2–3–4, reference: 1 = never; AOR per 1 unit increase)	1.13 (0.98–1.30)	0.100
Permanent aggression (ordinal 1–2–3–4, reference: 1 = never; AOR per 1 unit increase)	0.87 (0.71–1.06)	0.169
Cognitive impairment (cut-off ≥ 4) (Reference score = 3)	0.70 (0.56–0.88)	0.002

Values are cluster-adjusted odds ratios (95% confidence interval) and p-values.

R^2 ^= Pseudo R^2 ^by McFadden (1974).

AOR = adjusted odds ratio.

* A total of 154 residents without psychotropic medication prescription were excluded because of missing values.

Only few residents' characteristics were associated with psychotropic medication prescription. Cognitive impairment was inversely associated (AOR 0.70, 95% CI 0.56–0.88). In contrast, permanent restlessness was significantly associated with prescription of antipsychotic (AOR 1.47, 95% CI 1.33–1.64), anxiolytic (AOR 1.23, 95% CI 1.09–1.39), and hypnotic medication (AOR 1.29, 95% CI 1.11–1.49) as well as with prescription of any psychotropic medication (AOR 1.54, 95% CI 1.32–1.79). Legal guardian designated was inversely associated with antipsychotic medication prescription (AOR 0.66, 95% CI 0.50–0.86), as well as permanent inappropriate handling of things with antidepressant medication prescription (AOR 0.83, 95% CI 0.73–0.95).

Characteristics associated with psychotropic medication prescription and the different psychotropic medications, which turned out to be statistically significant at least within one of the final regression models with different outcomes, are displayed in Table 3 and Additional file 1.

## Discussion

This large study demonstrates that almost three-fourth of residents in nursing homes in Vorarlberg, Austria, have at least one prescription of a psychotropic medication.

The high prevalence of psychotropic medication prescription in our sample is comparable to results reported by earlier studies from other countries. For Dutch and Swedish nursing homes a prevalence of approximately 70% has been reported [32,33]. Only one Finish study reported a prevalence of 80% exceeding our finding [2]. This study determined a 20% prevalence of typical antipsychotics compared to almost 35% in our study. A recently published cross-sectional study covering data from five countries found pronounced variations between countries in prescription rates of antipsychotic medication from 11% in Hong Kong up to 38% in Finland [34].

Our study found a proportion of 35% of residents with at least one typical antipsychotic medication out of 46% of residents with any antipsychotic medication, a frequency that has never been reported before.

The shift in the prescription practice from conventional to atypical antipsychotics, as reported by Rapoport [35], has obviously not yet taken place in Austria. Since bedtime use prescription of typical antipsychotics was found to be common and prescription of hypnotics was low, suspicion can be raised upon possible inappropriate use of antipsychotics for treatment of sleeping disorders [36]. We have confirmed the finding by Alanen et al. (2006) that there is no significant difference in the frequency of antipsychotic medication prescription between residents with and without cognitive impairment [1].

In our study, antipsychotic medication prescription was significantly associated with the presence of restlessness. Whether these symptoms represent behavioural symptoms inadequately controlled by antipsychotics or whether they are induced by antipsychotics remains unknown. Associations should not be interpreted as causality. Nevertheless, the use of antipsychotics is not recommended for managing dementia-related wandering, pacing or repetitive vocalisations but only for behaviour potentially causing danger to the patient or others. In addition, the benefit of atypical antipsychotics is likely to be rather small despite a high risk of adverse effects [37]. Several other studies confirm the frequent prescription of antipsychotics to control behavioural and psychological symptoms in patients with dementia [1,38].

A publication by Hughes et al. suggests an association between high prescription rate of antipsychotic medication and a low staff/residents ratio [39]. This finding is not supported by our study, as we did not find an association between prescription prevalence and nursing staff/residents ratio. A recent study from Germany reported a lower rate of antipsychotic medication prescription despite comparable staff/resident ratio [3]. However, external evidence remains conflicting and indicates that other characteristics such as treatment variations and culture of care might be important as well [40,41].

The use of tricyclic antidepressants is not recommended in geriatric patients [42]. We found a prescription rate of 8% compared to 30.8% for SSRIs. Comparable studies reported significantly lower rates [3,18,43]. The inverse association between antidepressant medication prescription and male gender has also been reported in previous studies [2,18,44].

Since we did not assess duration of medication prescription, we cannot draw any conclusion about the magnitude of inappropriate medication according to the Beers Criteria or other criteria.

This is the first study analysing the prevalence of psychotropic medication prescription in Austrian nursing homes. We investigated an unselected study population consisting of 92% of all residents living in nursing homes in the Austrian federal state of Vorarlberg. The results of our study are likely to be transferable to other Austrian regions since nursing home characteristics and medical care delivered by general practitioners is comparable throughout the country [45]. In a previous study we demonstrated that the Austrian new legal act obligating nursing homes to report chemical restraints could not be executed due to lack of reporting standards on chemical restraints [23]. Still, this legal act could be a promising approach achieving a reduction of psychotropic medication. Therefore, joint efforts should be undertaken in order to develop an appropriate national reporting standard.

Our study has limitations. Data on medication prescribed "as required" were not included since no information on the frequency of administration was available. For data protection reasons, we were not able to assess residents' diagnoses. However, several studies have demonstrated discrepancies between mental health diagnoses and the use of psychotropic medications in nursing home residents [46,47]. Therefore, psychotropic medication use is not expected to be associated with diagnosis of BPSD or with major psychiatric diagnoses [1]. Also for data protection reasons, behavioural symptoms and cognitive impairment were assessed using proxy rating instruments rather than direct assessment. Validity of these instruments might be limited.

## Conclusion

This study is another piece of evidence indicating the ongoing overuse of psychotropic medication in nursing home residents. Our assumption that internationally published reports on adverse effects of psychotropic medication might have had an impact on prescription behaviour in Austrian nursing homes could not be confirmed.

There clearly is an urgent need to reduce and optimise psychotropic medication prescription in Austrian nursing homes.

## Competing interests

The authors declare that they have no competing interests.

## Authors' contributions

EM and GM initiated the study. GM and SK developed the study protocol. EM coordinated the study. EM and SK led analysis of data. BH performed the statistical analysis. All authors interpreted data. EM wrote the paper with major support by GM, BH wrote the statistical section. SK, BH and KP commented on paper drafts in all stages. All authors read and approved the final manuscript. EM is guarantor for the paper.

## Pre-publication history

The pre-publication history for this paper can be accessed here:



## Supplementary Material

Additional file 1 **Table 4**. Characteristics associated with prescription of antipsychotic, anxiolytic, hypnotic and antidepressant medication. Click here for file
