# Supplementary material for: Psychotropic medication use among nursing home residents in Austria: a cross-sectional study
Source: BMC Geriatr. 2009 May 21;9:18. doi: 10.1186/1471-2318-9-18 (PMC2697155; doi:10.1186/1471-2318-9-18)
Supplement: Additional file 1 — Table 4. Characteristics associated with prescription of antipsychotic, anxiolytic, hypnotic and antidepressant medication. [file 1471-2318-9-18-S1.doc]

Table 4: Characteristics associated with prescription of antipsychotic, anxiolytic, hypnotic and antidepressant medication.

| Characteristics | Antipsychotic medication  n=1690^*^  R^2^=0.069 | Anxiolytic medication  n=1690^*^  R^2^=0.031 | Hypnotic medication  n=1690^*^  R^2^=0.026 | Antidepressant medication  n=1690^*^  R^2^=0.036 |
| --- | --- | --- | --- | --- |
| Age  (Years, continuous variable; AOR per 1 year increase) | 0.99 (0.98-1.00), p=0.008 | 0.99 (0.98-1.00), p=0.061 | 1.01 (1.00-1.02), p=0.167 | 0.98 (0.97-0.99), p=0.004 |
| Male gender  (Reference: female) | 1.05 (0.87-1.28), p=0.599 | 0.62 (0.47-0.82), p=0.001 | 1.20 (0.87-1.65), p=0.257 | 0.60 (0.45-0.80), p=0.001 |
| Level of long-term care need ≥ 4  (Reference: 0-3) | 1.58 (1.16-2.14), p=0.003 | 0.93 (0.70-1.24), p=0.615 | 1.12 (0.83-1.49), p=0.462 | 1.47 (1.14-1.90), p=0.003 |
| Legal guardian designated  (Reference: no) | 1.52 (1.16-2.00), p=0.002 | 1.04 (0.80-1.36), p=0.751 | 0.71 (0.50-1.01), p=0.055 | 0.83 (0.68-1.02), p=0.077 |
| Fall during preceding 12 months  (Reference: no) | 1.12 (0.91-1.38), p=0.281 | 1.18 (0.95-1.48), p=0.134 | 1.04 (0.72-1.49), p=0.837 | 1.35 (1.07-1.70), p=0.012 |
| Permanent restlessness (ordinal 1-2-3-4, reference: 1=never; AOR per 1 unit increase) | 1.47 (1.33-1.64), p<0.001 | 1.23 (1.09-1.39), p=0.001 | 1.29 (1.11-1.49), p=0.001 | 1.01 (0.88-1.17), p=0.847 |
| Permanently handling things inappropriately (ordinal 1-2-3-4, reference: 1=never; AOR per 1 unit increase) | 1.11 (0.96-1.28), p=0.170 | 1.03 (0.89-1.21), p=0.673 | 0.93 (0.77-1.13), p=0.448 | 0.83 (0.73-0.95), p=0.006 |
| Permanent negative  (ordinal 1-2-3-4, reference: 1=never; AOR per 1 unit increase) | 0.96 (0.87-1.06), p=0.428 | 1.24 (1.08-1.42), p=0.002 | 1.14 (1.00-1.30), p=0.049 | 1.33 (1.20-1.48), p<0.001 |
| Permanent aggression  (ordinal 1-2-3-4, reference: 1=never; AOR per 1 unit increase) | 1.01 (0.84-1.21), p=0.939 | 0.80 (0.66-0.97), p=0.026 | 1.14 (0.91-1.43), p=0.261 | 0.85 (0.73-0.99),  p=0.032 |
| Cognitive impairment (cut-off > 4)  (Reference score ≤ 3) | 0.94 (0.70-1.25), p=0.661 | 0.61 (0.47-0.79), p<0.001 | 0.59 (0.40-0.88), p=0.009 | 0.81 (0.63-1.04), p=0.097 |

Values are cluster-adjusted odds ratios (95% confidence interval) and p-values.

R² = Pseudo R² by McFadden (1974).

AOR = adjusted odds ratio.

^*^ A total of 154 residents without psychotropic medication prescription were excluded because of missing values.
